# Editorial: Bridging the gap between the different pillars of tinnitus research

**DOI:** 10.3389/fnins.2025.1631559

**Published:** 2025-06-17

**Authors:** Alice L. Burghard, Adam Hockley, Elouise A. Koops, Joel I. Berger

**Affiliations:** ^1^Department of Neuroscience, University of Connecticut School of Medicine, Farmington, CT, United States; ^2^Institute of Neuroscience of Castilla y León (INCYL), University of Salamanca, Salamanca, Spain; ^3^Department of Radiology, The Athinoula A. Martinos Center for Biomedical Imaging, Massachusetts General Hospital, Harvard Medical School, Boston, MA, United States; ^4^Department of Neurosurgery, University of Iowa, Iowa City, IA, United States

**Keywords:** human studies, animal studies, tinnitus, patient care, tinnitus diagnosis, objective test for tinnitus, neural correlates of tinnitus

## Introduction

Subjective chronic tinnitus—the persistent perception of a sound in the absence of an exteroceptive stimulus—affects ~10% of the population (Bhatt et al., 2016). Despite several promising animal treatment studies, there are still no universally effective treatments in humans, wherein the focus is primarily on ameliorating the burden of tinnitus (contrasting with animal studies focusing on reducing the percept or neurophysiological correlates), while many of the computational models on the condition remain empirically untested. Thus, it is vital that we bridge the different pillars of tinnitus research to understand how to accurately assess and treat the condition.

**Figure 1:**
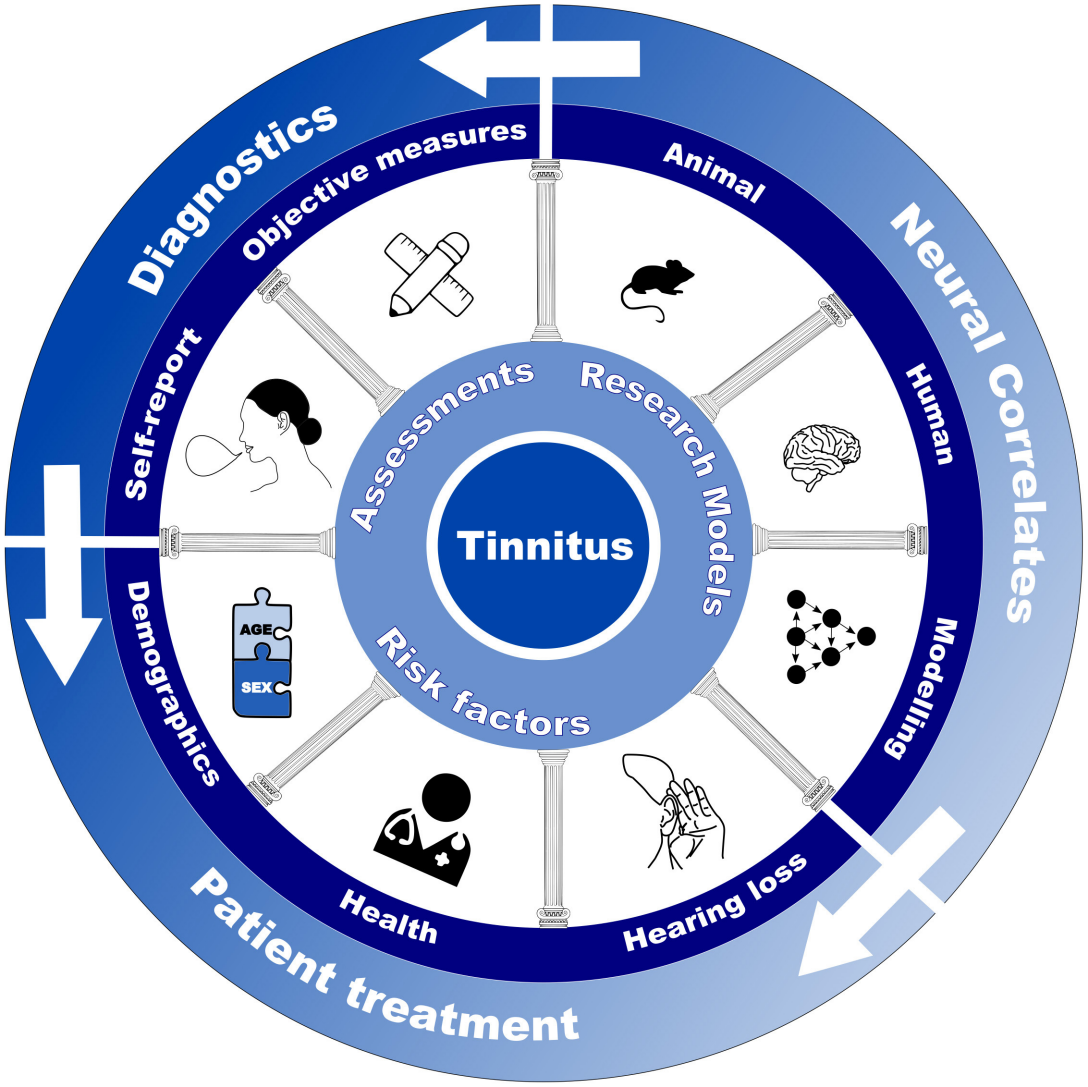
Schematic depicting the various pillars of tinnitus research. Figure created in Inkscape, including two icons generated in Biorender (https://BioRender.com/tn5pscs).

The current Research Topic was developed as an extension of a symposium that we and other tinnitus researchers hosted at the Association for Research in Otolaryngology Midwinter meeting in February 2023, which itself came about following discussions on existing gaps in tinnitus research. With this Research Topic, we aimed to bridge the gap between the pillars of tinnitus research. These pillars are outlined in Figure 1. By bringing various research strands together into one journal issue, we intend to reduce the separation between areas of research that should be complementary rather than separated. Researchers may then consume informative studies that they might otherwise consider falling outside of the scope of their own work.

In this Research Topic we collected six original research articles and one methodological article, all aimed at improving testing for tinnitus in humans or animals, understanding the neural bases of tinnitus, or developing and improving effective treatments options.

At the core of basic tinnitus research is having a solid model. Animal research brings its own difficulties, with established behavioral tests sometimes resulting in contradictory results in the same animal (Fabrizio-Stover et al., 2022). The articles presented here advance our understanding of models or create new tests for tinnitus. Ding et al. studied the neural mechanism of the Gap Prepulse Inhibition of the Acoustic Startle (GPIAS) tinnitus test and revealed that it has a different mechanism to the widely used prepulse inhibition test. Given that these tests are used to identify hearing status and tinnitus in animals, this is a crucial step to understanding changes in this phenomenon following noise exposure or drug administration. Wallace et al. built upon a previous use of the GPIAS test using 3D motion tracking of body markers (Berger et al., 2013) and optimized this methodology by measuring movement velocity. They also adapted motion tracking of the startle in the GPIAS paradigm to a mouse model, using back or tail markers to provide a reliable assessment of tinnitus. These improvements will allow wider use of the method in future studies.

The neural mechanisms of tinnitus are still a key area of study, with different hypotheses spanning the central auditory pathway (Hockley and Shore, 2023). Wake et al. recorded evoked activity changes in the auditory cortex of rats and studied how previous noise exposure would affect multi-unit activity and local field potentials. After noise exposure they behaviorally tested for tinnitus using GPIAS and for hyperacusis using PPI. Tonotopic remapping in the auditory cortex correlated with the behavioral measure of tinnitus, and cortical recruitment functions of multi-unit activity correlated with behavioral evidence of hyperacusis. Their data disentangled the neural correlates of tinnitus and hyperacusis in the auditory cortex.

One issue with the development of tinnitus treatments in humans is the lack of an objective test (Fabrizio-Stover et al., 2024). The development of an objective test would enable greater understanding of tinnitus mechanisms and may speed up treatment development in both animal and human research. Fabrizio-Stover et al. highlight a possible objective test for tinnitus in mice, utilizing auditory brainstem recordings in response to stimuli before and after long-duration sound (ABR_LDS_) and multi-unit recordings in the inferior colliculus (IC) to the same stimulus paradigm to reveal differences between noise exposed tinnitus and non-tinnitus mice.

In human tinnitus research, we need to take confounding factors into account as many individuals with tinnitus have concomitant hearing loss, which is especially prominent in older adults. Chen et al. performed a multi-center cross-sectional study focusing on the confounding factor of age-related hearing loss (ARHL). Importantly, by looking at older adults, they show that ARHL patients with tinnitus have lower hearing thresholds. Intriguingly, this is similar to what Fabrizio-Stover et al. showed, where tinnitus animals had ABR and multi-unit IC responses to the LDS paradigm that were more similar to unexposed animals than non-tinnitus animals, and also consistent with Koops et al. (2020) who demonstrated in humans that tonotopic maps of tinnitus individuals with hearing loss were more similar to controls compared to hearing loss individuals without tinnitus.

Ultimately, the reason we are invested in tinnitus research is to aid patients experiencing bothersome tinnitus and this warrants the translation of research outcomes into criteria that are useful in a clinical setting. The studies of Hoetink et al. and Lehóczky et al. aimed to provide research-informed criteria to facilitate clinical decision making. Hoetink et al. and Lehóczky et al. examine the factors that influence joint decision making of clinicians and patients to select treatment options for hearing loss and tinnitus. Several of these factors are reflected in baseline subjective report measures, such as tinnitus handicap scores, or in objective measures, such as audiometric thresholds. While the former is a uniquely human consideration in tinnitus (*i.e*. subjective report of tinnitus severity), it is plausible to assume that if there was a reliable objective measure of tinnitus in humans, such as those initially developed in animals, this could provide an important metric to measure treatment success. One of the test batteries developed in a study within this Research Topic could indeed represent such a test.

## Conclusion

The intended purpose of this Research Topic is to bridge the gaps between the different pillars that support tinnitus research, ultimately facilitating clinical decision making based on research-informed criteria. As a field, an emphasis on bridging these gaps means that studies are genuinely informed by one another, rather than separating into different paths. In doing so, we can then optimize approaches to characterizing tinnitus and ultimately inform the development of more effective treatment strategies.
